# Garden Snail Predatory Insects’ Modus Operandi Under Laboratory Conditions

**DOI:** 10.3390/insects15110865

**Published:** 2024-11-05

**Authors:** Giulia Murgia, Valentina Coroneo, Carlo Zuddas, Sara Maria Pani, Maria Paola Cogoni

**Affiliations:** 1 Istituto Zooprofilattico Sperimentale della Sardegna, 09125 Cagliari, Italy; 2 Department of Medical Sciences and Public Health, University of Cagliari, 09124 Cagliari, Italy

**Keywords:** snail farming, snail predatory beetle, *Helix aspersa*, *Cornu aspersum*, shell lesions

## Abstract

Heliciculture farms are susceptible to significant biotic issues that can impact snail breeding, among them, the entomofauna predation of snails. Predatory insects can cause damage to snail shells during predation, and sometimes, the specific type of damage may be characteristic of certain insect families or species. Under laboratory conditions, we analysed the predatory activity of the species *Silpha tristis* Illiger, 1798 (Coleoptera: Silphidae), *Ocypus olens* (Müller, 1764) (Coleoptera: Staphylinidae), *Carabus* (*Macrothorax*) *morbillosus constantinus* Kraatz, 1899 (Coleoptera: Carabidae), and *Lampyris sardiniae* Geisthardt, 1987 (Coleoptera: Lampyridae) against the gastropod *Cornu aspersum* (Müller, 1774) reared on snail farms located in the Sardinian region. The adult and larval stages of each species were tested, except for *L. sardiniae*, as only the larval stage preys upon snails. This study showed that among all of the species considered, only two insects were able to damage the shell of *C. aspersum: C. morbillosus constantinus* and *O.olens*. This may prove valuable in *C. aspersum* breeding for the recognition of specific lesions, even in the absence of the predator.

## Simple Summary

This preliminary study aims to shed light on the behaviour of some predatory entomofauna species found in the snail farms of Sardinia. We observed the interactions between snails and their natural predators in laboratory conditions. This approach enabled us to address a significant gap in the scientific literature by investigating whether specific types of shell damage could be characteristic of certain insect families or species.

## 1. Introduction

Snail farming is growing steadily worldwide, with a significant increase in the number of farms and production lines. In Italy, the demand for snails has risen considerably in recent decades for food consumption and use in the cosmetic, medical, and pharmaceutical sectors [1]. For this reason, snail farming has become an increasingly widespread agro-zootechnical activity that requires a deep understanding of production issues, animal welfare, and food safety. In Sardinia, snail farming has significant potential but is hindered by the lack of adequate research in this particular area. A more accurate knowledge of biotic challenges would allow for the implementation of more effective prevention and control strategies, significantly reducing economic losses. A previous study [2] highlighted how predation can pose a significant threat to the sustainability of farms. However, the lack of specific data on the farmed species and predatory insects present in Italy and Sardinia makes it challenging to develop adequate management protocols.

Climatic and biotic adversities are the main issues that affect snail breeding. Predators, such as entomofauna, can cause significant damage to snail farms. Sardinia hosts a high number of snail farms across several geographic areas, and in some of these farms, sampling has been carried out to understand the existing predatory entomofauna and explore the possibility of timely interventions in case of insect-related issues [2]. Previous studies in Sardinia [1] found that the most frequently sampled snail predator species were *Silpha tristis* Illiger, 1798 (Coleoptera: Silphidae), *Ocypus olens* Müller, 1764 (Coleoptera: Staphylinidae), *C. morbillosus costantinus* Kraatz, 1899 (Coleoptera: Carabidae), and *Lampyris sardiniae* Geisthardt, 1987 (Coleoptera: Lampyridae). Several studies explored the predatory behavior of species, primarily belonging to the Carabidae family [3,4,5,6], which Symonds (2004) defined as the main natural enemies of snails. Furthermore, other authors studied the predatory behavior of birds and mammals towards terrestrial gastropods [7]. Other studies focused on other families, such as Drilidae, or ecological associations between terrestrial molluscs and carabids [8,9]. However, these studies did not address the species examined in the present study, except for O. olens [10,11,12], which Orth (1975) had already identified as a predator of the snail *C. aspersum*. Notably, species such as *C. morbillosus costantinus* have been overlooked in these studies.

The species evaluated in the present study, whether polyphagous or helicophagous, cause the most problems for snail farms in Sardinia because they are attracted to both the snails and other insects present in this specific habitat [2].

**Figure 1:**
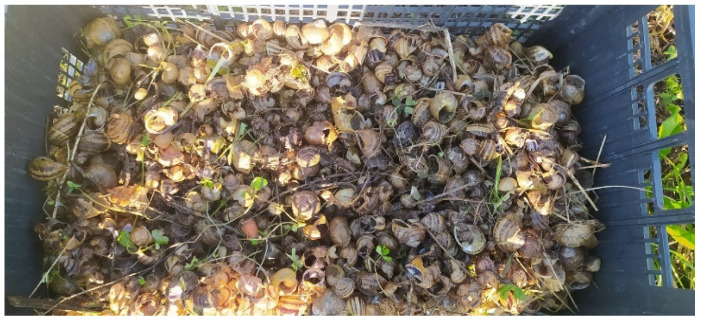
Dead snails with damaged shells found during the sampling of snail farms in Sardinia (photo by Giulia Murgia).

A snail’s shell serves as its main defence against predation. As a result, predators have developed techniques to overcome this barrier [3]. Additionally, the ability of snails to secrete mucous secretions poses another obstacle that predators must overcome. Natural snail antagonists often damage or break the shell, both from the outside and inside [3,4,5,6,7,8,9,10,11,12,13,14], or they attack the snail without damaging the shell. The discovery of snails with damaged shells and no traces of predators during site inspections (Figure 1) prompted the need to expand our knowledge of the predatory entomofauna sampled in Sardinia.

In this work, we observed the predatory behaviour of certain insects towards the gastropod *C. aspersum*. This study focused on the behaviour of *Staphylinid O. olens* at both the adult and larval stages, carabid *C. morbillosus costantinus* at the adult and larval stages, silphid *S. tristis* at the adult and larval stages, and *L. sardiniae* only at the larval stage. The aim was to enhance our understanding of the modus operandi of different predator species of *C. aspersum* and aid in the recognition of lesions on snail shells to implement timely containment measures and limit snail mortality in snail farms.

## 2. Materials and Methods

### 2.1. Species of Coleoptera Involved in This Study

*O. olens* belongs to the family Staphylinidae; it is the only species for which studies describing its predatory behaviour are available. Information on its helicophagous habits in both the larval and adult stages has been previously described [10,11,12]. The adult stage of this species has an elongated, robust, black body with a large head and massive toothed mandibles on the inner edge. It can reach around 32 mm in length, making it the largest species within the family Staphylinidae [15]. It is found throughout the Palaearctic region and is widespread in Italy, including the islands [16]. It typically lives in several habitats, especially in damp woods, grasslands, abandoned areas, and gardens, and can often be found during the day under stones or plant material. This species is a polyphagous predator, consuming plants, invertebrates, and animal carcasses in both its adult and larval stages [12,13,14,15,16,17]. In Britain, it actively preys upon *C. aspersum* (*Helix aspersa*) and *Helix pomatia* L. [10,11,12,13,14,15,16,17,18]. When disturbed, this Staphylinidae assumes a characteristic posture, raising the end of its abdomen and opening its jaws, which are adept at grasping prey. When it feels threatened, it secretes a repellent substance from two small glands located on its abdomen [17].

*C. morbillosus constantinus* is mainly found in North Africa, Sardinia, and Lampedusa. It is a large beetle, ranging in size from 25 to 35 mm, copper-bronze in colour, with brighter shades on the edge of the pronotum and at the margins of the elytra; the body is oval-shaped, with a flattened head and pronotum, and it has robust sickle shaped mandibles [16]. Like many carabids, it lives mainly in rural areas where it preys upon snails, caterpillars, and earthworms. While it also consumes plants, it is considered a species that primarily feeds on snails [19].

*S. tristis* is commonly found throughout central and southern Europe. This species is approximately 13–14 mm in length and is black in colour with a wide, flattened body and head and short mandibles. It has necrophagous habits and actively preys upon the eggs and larvae of necrophilous species that lay their eggs on corpses or animal carcasses [20,21,22]; it can also prey upon terrestrial gastropods [2,3,4,5,21].

**Figure 2:**
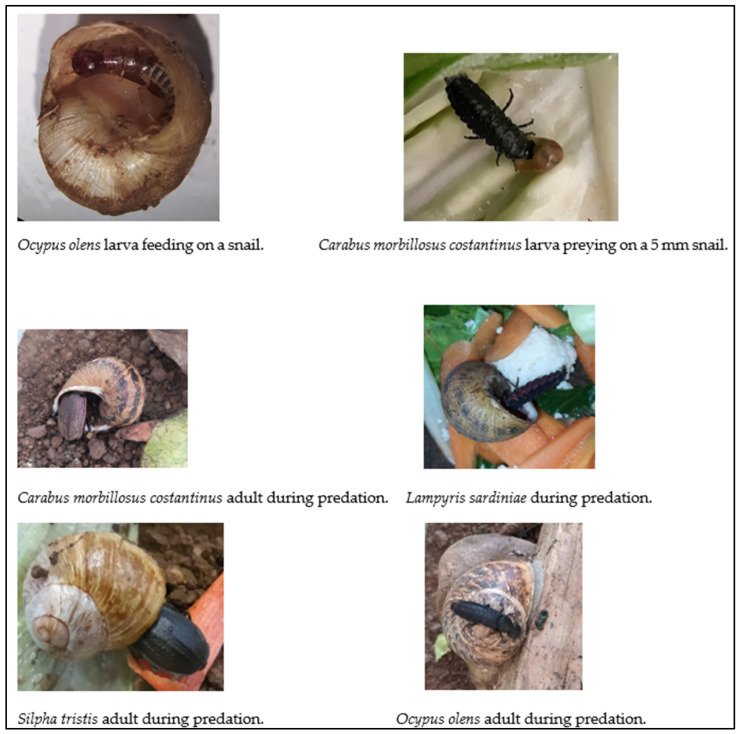
Species of Coleoptera involved in this study during predatory activities (photos by Giulia Murgia).

*L. sardniae* is endemic to Sardinia. Its larval stage measures around 20–25 mm, being velvety black in colour with fuchsia or magenta lateral bands, which appear almost fluorescent. It is commonly found in holm oak and cork oak woods, hedges, uncultivated areas, and hygrophilous environments where its prey, mainly consisting of gastropod molluscs, are found. The adult male of this species has a glycophagous diet (sugar-rich foods such as nectar and plant fluids), while the female is entomophagous [23,24]. Figure 2 shows the species included in the study during their predatory activities.

### 2.2. Study Design

**Figure 3:**
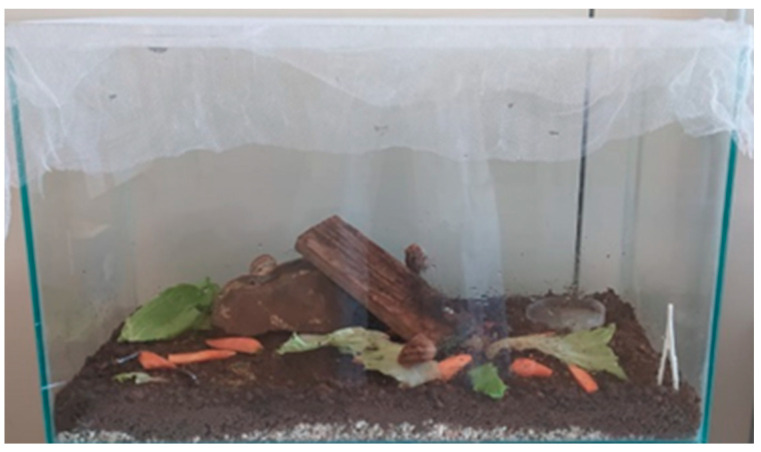
Example of the terrarium used for the experiment (photo by Giulia Murgia). The experimental terrarium had a fine-mesh polyester screen (0.25 mm) top to prevent the entry of opportunistic insects. Environmental conditions: temperature 23 °C and relative humidity 80%. Substrate composed of topsoil, wood, and fine gravel (0.5 mm) to promote drainage. Nutrition: plant-based substrate.

In this experiment, we set up two identical terrariums that replicated the snail breeding habitat (see Figure 3). Both terrariums were used to speed up the experiments. However, only direct observations of predation-induced mortality were recorded and snails that died outside of observation periods were replaced with snails of the same size.

Within each terrarium, three specimens of a predatory species were introduced in a cyclical manner together with five snails of the species *C. aspersum* (Table 1a,b). We studied the adult stages of the species *S. tristis, O. olens*, and *C. morbillosus constantinus*, along with five snails of each size. In each trial, the snails were 30–35, 18–22, and 4–6 mm in size, plus the egg stage; each category was evaluated for seven days. Adult snails can be distinguished from juveniles by the thickening of the shell in the area of the opening (peristome). We also evaluated the larval stages of the abovementioned species and *L. sardiniae*, again for seven days per species and per snail size. We conducted two additional replicates by replacing the preyed-upon snails with others of the same size (five for each trial) (Table 1a,b) and replacing the insects to increase the sampling variability within the same species. Each experimental trial included a group of three conspecific insects, which was replaced with a new set of three after each trial.

A random sex ratio of predatory insects was maintained, with an estimate of approximately 2/3 being female. This choice was based on the differences observed in a previous study that analysed the insect species collected from Sardinian snail farms [2], where the sampled predatory insects were predominantly female. Each group of predators had about three days to adapt to the captive conditions before the experiment started. During this time, the insects and snails were kept in separate environments. The experimental period lasted approximately 13 months, from September 2021 to October 2022.

The terrariums measured 60 cm in length, 30 cm in width, and 35 cm in height, and were set up with a bottom layer of soil that had been sieved and sterilised beforehand. The sieve had an 8-mesh screen with a clear aperture of 2.36 mm. The residue that passed through the mesh was the material used in our analysis. The terrariums were supplied with enough water daily to maintain the necessary humidity level. The average temperature was approximately 23 °C, and we maintained a high humidity level (~80%) by spraying water several times a day. The snails and insects were provided with plant nutrient substrates. Shelters such as stones and wooden slats, sterilized by boiling and freezing respectively, were introduced to provide protection for the insects and recreate a habitat similar to a snail farm. All terrarium materials were field-collected, analyzed, and cleaned to prevent contamination.

To study the modus operandi of these zoophagous species, we conducted visual observation for about 8 h a day in dimmed light conditions. This setting facilitated the predatory activity of the entomological species upon the gastropods.

### 2.3. Statistical Analysis

We applied the Chi-square test to verify whether there was a significant difference between the number of snails preyed upon by each predator using the aggregated data from the three trials. We also applied a Chi-square test to verify whether there was a significant difference between the number of snails of different sizes preyed upon by each predator (individually). We applied the Fisher’s exact test (when 20% of the cells of the contingency matrix had expected frequencies <5) to verify individually for each predator and each snail size whether there was a significant difference in the frequency of snails preyed upon with or without shell damage. *p*-values < 0.05 were considered significant. The software employed to carry out the analysis was Microsoft Excel (Microsoft Corporation, 2018).

## 3. Results

### 3.1. General Results

The difference between the frequency of snails preyed upon by each predator was statistically significant (χ^2^ (6, N = 420) = 13.322, *p* < 0.05), and the analysis of residuals showed that *S. tristis* adults (adjusted residual 2.886; −2.886; *p* < 0.01) tended to prey upon more snails.

Furthermore, the species *S. tristis* (larva and adult) was the primary predator of *C. aspersum* eggs (χ^2^ (1, N = 105) = 23.133, *p* < 0.01), with a frequency significantly higher than expected (adjusted residual 4.810; *p* < 0.01) when compared to all the other predators combined.

Although adult *C. morbillosus costantinus* and adult *O. olens* were the only two predators that caused shell damage to *C. aspersum* with diameters between 20 and 35 mm, we did not find a significant difference between the frequencies of the two categories of dead snails (no shell damage/shell damage) when examining each predator individually (see Table S1).

### 3.2. O. olens Adult and Larval Stages vs. C. aspersum at Different Growth Stages

Adult *O. olens* preyed upon adult *C. aspersum*, between 30 and 35 mm in size, in a predictable sequence. The attack began with repeated bites to the extended head. When the snail was inactive and completely retracted inside its shell, the insect continuously tried to enter the shell. As a defence mechanism, the snail produced an abundant mucous secretion, forcing the insect to exit the shell and repeatedly clean its legs and body. It was observed that the attack on the snail lasted about two hours until it was completely dehydrated and died. This species never pierced or crushed shells of this size (Table 2, Table 3 and Table S2).

**Figure 4:**
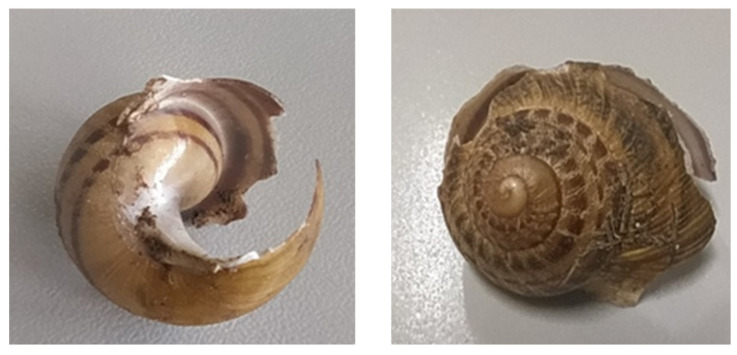
Shell damage of an 18–22 mm shell caused by adult *Ocypus olens* (photo by Giulia Murgia).

Snails with a diameter of 18–22 mm were attacked in a similar manner. However, in five cases, unlike the larger snails, the shells were crushed from the peristome to the shell apex, following the intrasutural line where the shell is more fragile (Figure 4) (Table 2, Table 3 and Table S3). The mode of predation on 4–6 mm snails was very similar to that used on larger snails, except that the shells were always completely crushed (Table 2, Table 3 and Table S4). Of the fifteen eggs in the three trials, only four were preyed upon (Table 2, Table 3 and Table S5).

The larval stage of *O. olens* used the same method of attack, albeit with considerable difficulty. In fact, snails 30–35 mm in size were never killed by the larval stage of *O. olens* despite repeated attacks. *O. olens* larvae preyed on 18–22 mm snails with a significantly higher frequency (χ^2^ (3; N = 60) = 28.938, *p* < 0.01) than those with other shell sizes.

No significant difference between the frequency of predation of different snail sizes was found for *O. olens* adults. The shells of 20 mm snails were never damaged, while four shells with a diameter of 46 mm were crushed. Both adult and larval *O. olens* would feed about 12 h after the snail’s death, attracted by the strong odour of the decomposing snail.

### 3.3. C. morbillosus Costantinus Adult and Larval Stages vs. C. aspersum at Different Growth Stages

**Figure 5:**
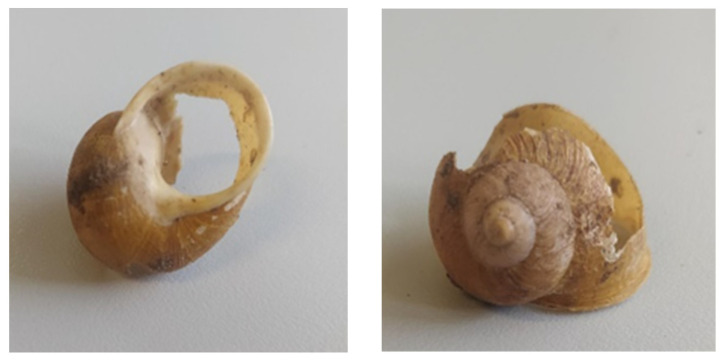
Shell damage of a 30–35 mm shell caused by adult *Carabus morbillosus costantinus* (photo by Giulia Murgia).

In the first phase of the experiment, adult snails 30–35 mm in size and *C. morbillosus* (at the adult stage) were used. After three days without feeding, except for scraping the fleshy part of some apples, the carabids started to prey upon the snails. Initially, the carabid attempted to turn the snail by placing the opening upwards and repeatedly biting it. The snail, in defence, began to produce mucous secretions. Then, the carabid tried to scrape the shell from the edge opening, trying to pierce it. Eventually, the carabid managed to break the shell in the intrasutural area, which is the most fragile area of the shell, located immediately after the peristome border (typical of adult snails) (Figure 5). Fourteen out of fifteen snails were preyed upon by *C. morbillosus costantinus*. Ten of these had their shells damaged, while four snails were consumed without shell damage (Table 2, Table 3 and Table S2). All the snails killed were devoured after death.

**Figure 6:**
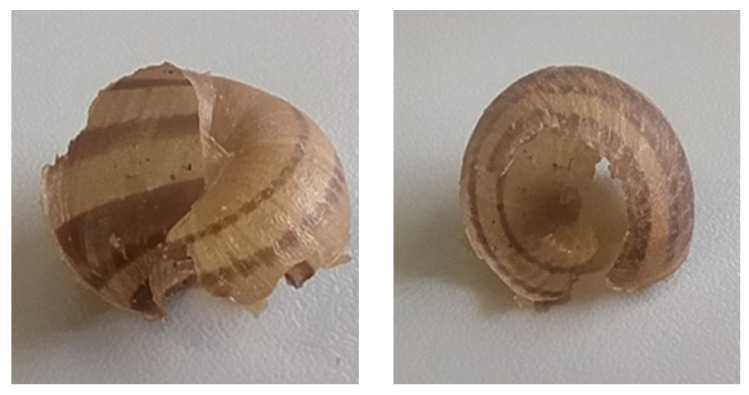
Shell damage of an 18–22 mm shell caused by adult *Carabus morbillosus costantinus* (photo by Giulia Murgia).

This modus operandi was observed both on adult snails with a thickened lip and diameter of 30–35 mm and snails with a diameter of 18–22 mm. Of the latter, eight out of fifteen snails were killed, and seven of those had damaged shells, starting from the aperture and continuing along the intrasutural line (Figure 6). Newly hatched 4–6 mm snails were also tested, and only one snail was preyed upon in the three trials, with its shell completely crushed. These predators never preyed upon eggs.

The mode of predation of larvae was similar to that of adults. They repeatedly bit the snail’s ocular tentacles and head, leading the snail to produce slime. Then, when the snail retracted into its shell, the larvae positioned the opening of the shell upwards and continued to stress the snail. Once deceased or exhausted, the snail was immediately devoured. The larval stage of this species never damaged 30–35 mm and 18–22 mm snail shells, nevertheless causing the death of 9 and 12 specimens, respectively. However, it caused shell damage in 9 out of the 11 4–6 mm snails that were killed. The egg stage was never preyed upon.

*C. morbillosus costantinus* larvae showed a statistically significant difference between the frequency of snails preyed upon in each different size group (χ^2^ (3; N = 60) = 23.719, *p* < 0.01); namely, they tended to prey upon 18–20 mm snails (adjusted residual 2.656; *p* < 0.01) and 5 mm snails (adjusted residual 2.066; *p* < 0.05) and not upon eggs (adjusted residuals −4.426; *p* < 0.01). Likewise, *C. morbillosus costantinus* adults showed a statistically significant difference between the frequency of snails preyed upon in each different size group (χ^2^ (3; N = 60) = 36.310, *p* < 0.01), tending to prey upon 30–35 mm snails (adjusted residual 5.059, *p* < 0.01) and not upon 4–6 mm snails (adjusted residual −2.913, *p* < 0.01) and eggs (adjusted residual −3.526, *p* < 0.01).

### 3.4. S. tristis Adult and Larval Stages vs. C. aspersum at Different Growth Stages

The species *S. tristis,* at the adult stage, exhibited the same initial behaviour as the previously mentioned species but never damaged the snail shells. In fact, during the experimentation, we observed that the *S. tristis* species never managed to consume the prey completely. Adult *S. tristis* preyed upon the greatest number of snails (37 specimens in total); (χ^2^ (6, N = 420) = 13.322, *p* < 0.05).

*S. tristis* larvae showed a significant difference between the frequency of preying upon snails across different size groups (χ ^2^ (3; N = 60) = 13.920, *p* < 0.01), tending to prey more upon 4–6 mm snails (adjusted residual 2.268; <0.05) and not upon 30–35 mm snails (adjusted residual −3.175; <0.01). On the other hand, *Sipha tristis* adults tended to prey more upon 18–22 mm snails (adjusted residual 2.300; *p* < 0.05) and not upon 4–6 mm snails (adjusted residual −3.833; *p* < 0.01).

### 3.5. L. sardiniae Larval Stage vs. C. aspersum at Different Growth Stages

*L. sardiniae* exhibited the same mode of attack as the other species. It preyed upon 18–22 mm, 30–35 mm, and 4–6 mm snails, and killed twelve, six, and eight specimens, respectively. There was a significant difference in the frequency of snails preyed upon by *L. sardiniae* larvae in each different size group (χ ^2^ (3; N = 60) = 20.362, *p* < 0.01). Specifically, they tended to prey more upon 18–22 mm snails (adjusted residual 3.309; *p* < 0.01) and not upon eggs (adjusted residual −3.911; *p* < 0.01). Shell crushing occurred in six of the eight 4–6 mm preyed-upon snails, while the other two snails were consumed without shell damage. In one case, *L. sardiniae* larvae damaged an 18–22 mm shell, making a single hole in the intrasutural line.

### 3.6. Shell Lesions

Table 4 shows a detailed description of the different types of shell lesions associated with the predators more likely to cause them.

## 4. Discussion

All observed species, after a brief search, detected their prey and used their antennae to examine the shell, the snails’ primary defence tool. The predators then attempted repeated attacks, often targeting the snail’s ocular tentacles first. This caused the snail to use its second defensive mechanism, which is the production of slime. The continuous production of slime led to the snail experiencing dehydration and exhaustion, eventually leading to its death. In this study, the most preyed upon snails were those with a diameter of 18–22 mm (i.e., no thickened lip), while the egg stage was the least preyed upon. All predatory species, both in their adult and larval stages, showed no significant differences in their ability to prey upon *C. aspersum* eggs. Adult *S. tristis* was the species that preyed upon the most snails, showing no difference in preference between 30–35 mm and 18–22 mm snails. The 30–35 mm snails were more frequently preyed upon by the adult stages of *C. morbillosus costantinus* and *S. tristis* than *O. olens*. The 18–22 mm snails were the most preyed upon by all stages and predator species. *O. olens* and *C. morbillosus costantinus* at the larval stage preyed upon the highest number of 4–6 mm snails without showing any differences in behaviour.

The beetle *C. morbillosus costantinus* caused the shell to break, starting from the shell opening, while feeding. The breaking continued along the intrasutural line where the shell is more fragile. The same operation was performed on snails with a thickened lip after the shell was damaged. On the other hand, snails 4–6 mm in size were shattered completely. It is interesting to note that a recent study [25] has shown that several generalist carabid species can cause characteristic damage to snail shells; however, *C. morbillosus costantinus* was not included in this analysis. The damage found on *C. aspersum* by *C. morbillosus costantinus* and described as ‘spiral grooves’ by Němec & Horsák and Millar & Waite [3,14] suggests that this species, not classified as helicphagous, might also exhibit similar predatory behaviors. The species *O. olens* only crushed the shell of snails with a diameter up to 18–22 mm. The shell of snails in this size range is more fragile, resulting in it being crushed from the opening to the apex. The only species that never shattered a shell was *S. tristis* because of its short mandibles. *S. tristis* partially consumed its prey, as it was unable to completely take the snails out of their shell or penetrate the shell, unlike the other species that totally consumed their prey. While adult *S. tristis* were not able to damage snail shells, they were still capable of killing a significant number of snails, representing an important issue in snail farming [2]. The larval stages had no difficulty entering the shell to entirely consume their prey, most likely due to their slender shape.

As mentioned previously, the predators of snail farms may also include small vertebrates that damage the shell to feed upon snails. Rodents seem to be attracted to small gastropods, probably because larger shells require more time and strength to break [26]. However, the type of lesion produced on the shell by adult *C. morbillosus costantinus* and adult *O. olens* differs significantly from the damage caused by birds and rodents [7,8,9,10,11,12,13,14,15,16,17,18,19,20,21,22,23,24,25,26]. Furthermore, birds are not a major threat to snails since they mainly feed upon beetles and other insects rather than snails [27,28]. The experimentation with the species *C. morbillosus costantinus* and *O. olens* provided species-specific patterns of shell shattering in *C. aspersum*, confirming the findings of a previous study in Sardinian snail farms [2]. The present study provides a valuable contribution to the field of the recognition of shell lesions/predatory insects even in the absence of the predators themselves. These findings could be useful not only for preventing and reducing snail mortality in snail farms, but also for controlling harmful gastropods in agriculture using native predator species.

## Figures and Tables

**Table 1:** (**a**) Experimental design: In the first trial, 5 snails of the species *Cornu aspersum* were introduced into the terrarium together with 3 predatory insects of a specific species for 7 days; subsequently, in the second trial, another 3 predatory insects of the same species were introduced together with 5 other snails of the same size and monitored for 7 days. The same experimental procedure was repeated in the third trial. This experimental design was applied to 4 different species of predatory beetles and repeated for each developmental stages of *Cornu aspersum* snails; (**b**) Species and developmental stage of the beetles used in the experiment and size of *Cornu aspersum* snails.

(**a**)				
**Trial 1**	**Trial 2**		**Trial 3**	
Day 1–7	Day 8–14		Day 15–21	
N = 3 Predatory insects + N = 5 *Cornu aspersum*	N = 3 Predatory insects + N = 5 *Cornu aspersum*		N = 3 Predatory insects + N = 5 *Cornu aspersum*	
(**b**)				
**Species and Number of Individuals** **Introduced into the Terrarium**	Trial 1–trial 2–trial 3 7d + 7d + 7d	Trial 1–Trial 2–Trial 3 7d + 7d + 7d	Trial 1–Trial 2–Trial 3 7d + 7d + 7d	Trial 1–Trial 2–Trial 3 7d + 7d + 7d
N = 9 *Ocypus olens* larvae	N = 15 *Cornu aspersum* 30–35 mm	N = 15 *Cornu aspersum* 18–22 mm	N = 15 *Cornu aspersum* 4–6 mm	N = 15 *Cornu aspersum* eggs
N = 9 *Ocypus olens* adults				
N = 9 *Carabus morbillosus costantinus* larvae				
N = 9 *Carabus morbillosus costantinus* adults				
N = 9 *Silpha tristis* larvae				
N = 9 *Silpha tristis* adults				
N = 9 *Lampyris sardiniae* larvae				

**Table 2:** Cumulative number of *Cornu aspersum* preyed upon by each predator.

	*Cornu aspersum*	*Ocypus olens* Larva	*Ocypus olens* Adult	*Carabus morbillosus costantinus* Larva	*Carabus morbillosus costantinus* Adult	*Silpha tristis* Larva	*Silpha tristis* Adult	*Lampyris sardiniae* Larva
**TRIAL 1**	30–35 mm	0	1	2	5	1	5	3
	18–22 mm	3	3	3	2	3	4	3
	4–6 mm	4	2	4	0	2	0	4
	eggs	0	0	0	0	1	2	0
**TRIAL 2**	30–35 mm	0	2	4	5	0	4	2
	18–22 mm	4	2	5	5	4	4	5
	4–6 mm	5	2	5	1	5	2	2
	eggs	1	3	0	0	2	4	0
**TRIAL 3**	30–35 mm	0	2	3	4	0	3	1
	18–22 mm	1	2	4	1	2	5	4
	4–6 mm	3	3	2	0	3	1	2
	eggs	0	1	0	0	2	3	0
	**Total for predator**	**21**	**23**	**32**	**23**	**25**	**37**	**26**

**Table 3:** Cumulative number of *Cornu aspersum* preyed upon by each predator divided by size.

	*Cornu aspersum*			
Predator	30–35 mm	18–22 mm	4–6 mm	Eggs
*Ocypus olens* larva	0	8	12	1
*Ocypus olens* adult	5	7	7	4
*Carabus morbillosus costantinus* larva	9	12	11	0
*Carabus morbillosus costantinus* adult	14	8	1	0
*Silpha tristis* larva	1	9	10	5
*Silpha tristis* adult	12	13	3	9
*Lampyris sardiniae* larva	6	12	8	0

**Table 4:** Details of the different types of shell lesions associated with the predators that are more/less likely to cause them.

	Predators	
Type of Lesion	Likely	Unlikely
Shell not broken and no lesions	*Silpha tristis* adult *Silpha tristis* larva *Carabus* (*Macrothorax*) *morbillosus constantinus* larva *Ocypus olens* larva *Lampyris sardiniae* larva	*Carabus* (*Macrothorax*) *morbillosus constantinus* adult *Ocypus olens* adult
Adult shell (30–35 mm) broken behind the lip in the intersutural area	*Carabus* (*Macrothorax*) *morbillosus constantinus* adult	*Silpha tristis* adult *Silpha tristis* larva *Carabus* (*Macrothorax*) *morbillosus constantinus* larva *Ocypus olens* larva *Lampyris sardiniae* larva *Ocypus olens* adult
Juvenile shell (18–22 mm) broken from the aperture edge along the intrasutural area	*Carabus* (*Macrothorax*) *morbillosus constantinus* adult	*Silpha tristis* adult *Silpha tristis* larva *Carabus* (*Macrothorax*) *morbillosus constantinus* larva *Ocypus olens* larva *Ocypus olens* adult *Lampyris sardiniae* larva
Juvenile shell (18–22 mm) broken from the aperture edge to the spire	*Ocypus olens* adult	*Silpha tristis* adult *Silpha tristis* larva *Carabus* (*Macrothorax*) *morbillosus constantinus* larva *Ocypus olens* larva *Lampyris sardiniae* larva
Juvenile shell shattered (under 6 mm)	*Ocypus olens* larva *Ocypus olens* adult *Carabus morbillosus costantinus* larva *Carabus morbillosus costantinus* adult *Lampyris sardiniae* larva	*Silpha tristis* larva *Silpha tristis* adult

## Data Availability

The original contributions presented in the study are included in the article; further inquiries can be directed to the corresponding author/s.

## Supplementary Materials

The following supporting information can be downloaded at: https://www.mdpi.com/article/10.3390/insects15110865/s1, Table S1: Fisher’s exact tests results for each comparison (dead snails: no shell damage/shell damage) of each predator and each prey size. Predators that caused at least one death through shell damage are highlighted in blue and green: predators that caused the higher number of deaths with shell damage are highlighted in blue; predators that caused more deaths without shell damage are highlighted in green. NA: no snails of the size considered were preyed upon by the predator; Table S2: *Cornu aspersum* 30–35mm: details of the number of snails preyed upon by each predator with/without shell damage; Table S3: *Cornu aspersum* 18–22 mm: details of the number of snails preyed upon by each predator with/without shell damage; Table S4: *Cornu aspersum* 4–6 mm: details of the number of snails preyed upon by each predator with/without shell damage; Table S5: Number of *Cornu aspersum* eggs preyed upon by each of predator.
