# Supplementary material for: Garden Snail Predatory Insects’ Modus Operandi Under Laboratory Conditions
Source: Insects. 2024 Nov 5;15(11):865. doi: 10.3390/insects15110865 (PMC11594771; doi:10.3390/insects15110865)
Supplement: Supplementary file 1 [file insects-15-00865-s001.zip › insects-3257163-supplementary.pdf]

SUPPLEMENTAL MATERIALS

Tab S1. Fisher's exact tests results for each comparison (dead snails: no shell damage/shell damage) of each predator and each prey size. Predators that caused at least one death through shell damage are highlighted in blue and green: predators that caused the higher number of deaths with shell damage are highlighted in blue; predators that caused more deaths without shell damage are highlighted in green. NA: no snails of the size considered were preyed upon by the predator.

***Cornu aspersum* 30 – 35 mm**	
**Predator**	**P-value**
Ocypus olens larva	**NA**
Ocypus olens adult	p=0.0625
Carabus morbillosus costantinus larva	p=0.0039
Carabus morbillosus costantinus adult	p=0.1795
Silpha tristis adult	p=0.0004
Silpha tristis larva	p=1.0000
Lampyris sardiniae larva	p=0.0312
***Cornu aspersum* 18 – 22 mm**	
Ocypus olens larva	p=0.0078
Ocypus olens adult	p=0.4531
Carabus morbillosus costantinus larva	p=0.0004
Carabus morbillosus costantinus adult	p=0.0703
Silpha tristis adult	p=0.0039
Silpha tristis larva	p=0.0002
Lampyris sardiniae larva	p=0.0063
***Cornu aspersum* 4 – 6 mm**	
Ocypus olens larva	p=0.3876
Ocypus olens adult	p=0.4531
Carabus morbillosus costantinus larva	p=0.0654
Carabus morbillosus costantinus adult	p=1.0000
Silpha tristis adult	p=0.0019
Silpha tristis larva	p=0.2500
Lampyris sardiniae larva	p=0.2890

Tab S2. *Cornu aspersum* 30 – 35mm: details of the number of snails preyed upon by each predator with/without shell damage.

***Ocypus olens* larva**

***Cornu aspersum* 30 – 35mm**	No shell damage	Shell damage	Total
Dead	0	0	0
Alive	15	0	15
Total	15	0	15

***Ocypus olens* adult**

	No shell damage	Shell damage	Total
Dead	5	0	5
Alive	10	0	10
Total	15	0	15

***Carabus morbillosus costantinus* larva**

	No shell damage	Shell damage	Total

Dead	9	0	9
Alive	6	0	6
Total	15	0	15

Carabus morbillosus costantinus **adult**

	No shell damage	Shell damage	Total
Dead	4	10	14
Alive	1	0	1
Total	5	10	15

Silpha tristis **larva**

	No shell damage	Shell damage	Total
Dead	1	0	1
Alive	14	0	14
Total	15	0	15

Silpha tristis **adult**

	No shell damage	Shell damage	Total
Dead	12	0	12
Alive	3	0	3
Total	15	0	15

Lampyris sardiniae **larva**

	No shell damage	Shell damage	Total
Dead	6	0	6
Alive	9	0	9
Total	15	0	15

Tab S3. *Cornu aspersum* 18 - 22 mm: details of the number of snails preyed upon by each predator with/without shell damage.

Ocypus olens **larva**

Cornu aspersum **18 – 22 mm**	No shell damage	Shell damage	Total
Dead	8	0	8
Alive	7	0	7
Total	15	0	15

Ocypus olens **adult**

	No shell damage	Shell damage	Total
Dead	2	5	7
Alive	8	0	8
Total	10	5	15

Carabus morbillosus costantinus **larva**

	No shell damage	Shell damage	Total
Dead	12	0	12
Alive	3	0	3
Total	15	0	15

Carabus morbillosus costantinus **adult**

	No shell damage	Shell damage	Total
Dead	1	7	8
Alive	7	0	7
Total	8	7	15

***Silpha tristis* larva**

	No shell damage	Shell damage	Total
Dead	9	0	9
Alive	6	0	6
Total	15	0	15

***Silpha tristis* adult**

	No shell damage	Shell damage	Total
Dead	13	0	13
Alive	2	0	2
Total	15	0	15

***Lampyris sardiniae* larva**

	No shell damage	Shell damage	Total
Dead	11	1	12
Alive	3	0	3
Total	14	1	15

Tab S4. *Cornu aspersum* 4 - 6 mm: details of the number of snails preyed upon by each predator with/without shell damage.

Ocypus olens larva

Cornu aspersum 4-6 mm	No shell damage	Shell damage	Total
Dead	8	4	12
Alive	3	0	3
Total	11	4	15

Ocypus olens adult

	No shell damage	Shell damage	Total
Dead	2	5	7
Alive	8	0	8
Total	10	5	15

Carabus morbillosus costantinus larva

	No shell damage	Shell damage	Total
Dead	2	9	11
Alive	4	0	4
Total	6	9	15

Carabus morbillosus costantinus adult

	No shell damage	Shell damage	Total
Dead	0	1	1
Alive	14	0	14
Total	14	1	15

Silpha tristis larva

	No shell damage	Shell damage	Total
Dead	10	0	10
Alive	5	0	5
Total	15	0	15

Silpha tristis adult

	No shell damage	Shell damage	Total
Dead	3	0	3

Alive	12	0	12
Total	15	0	15

Lampyris sardiniae larva

	No shell damage	Shell damage	Total
Dead	2	6	8
Alive	7	0	7
Total	9	6	15

Tab S5. Number of *Cornu aspersum* eggs preyed upon by each of predator.

Predator	***Cornu aspersum* eggs**
Ocypus olens larva	1
Ocypus olens adult	4
Carabus morbillosus costantinus larva	0
Carabus morbillosus costantinus adult	0
Silpha tristis larva	5
Silpha tristis adult	9
Lampyris sardiniae larva	0
